# Living with a loved one’s mental health issue: Recognizing the Lived Experiences of Military Spouses

**DOI:** 10.1371/journal.pone.0336295

**Published:** 2025-11-07

**Authors:** Emma Senior, Amanda Clarke, Gemma Wilson-Menzfeld

**Affiliations:** Department of Nursing, Midwifery and Health, Northumbria University, Newcastle-Upon-Tyne, United Kingdom; Caleb University, NIGERIA

## Abstract

Limited evidence surrounds the lived experiences of military spouses whose partner has mental health issues. This lack of evidence may be due to factors such as global austerity, underfunding of armed forces, and inadequate healthcare systems. As a result, family members—especially spouses—often end up being the primary caregivers for their military partners with mental health issues. The study used a qualitative, biographical methodology, collecting data through life stories. Two face-to-face semi-structured interviews took place with nine military spouse recruited through military spouse networks and snowballing. Lieblich et al.’s (1998) framework provided analytical pluralism, which allowed for both narrative and thematic analysis. Stories are presented in the stages ‘in the beginning’, changing times’ and ‘this is me’. Thematic analysis identified six overarching categories; Living with disruption, living in the midst of it all, It isn’t enough, seeking support, Diagnosis and treatment, Living alongside. Whilst the first of its kind in the UK, this biographical study advances both national and global understanding of military spouse experiences in the context of mental health. Both the stories and the categories indicate that living with a serving partner who has mental health issues is a complex journey marked by both struggle and growth. A uniqueness arising from this study highlights the period leading up to a mental health diagnosis, emphasising the prolonged emotional and psychological strain experienced by military spouses before any formal recognition of mental illness in their serving partner. The study adds a new dimension to understanding the emotional toll on military spouses and underscores the importance of early recognition and support. While participants faced emotional detachment and feelings of invisibility, they also identified gains in resilience and strengthened relationships. Through the convergence of the narrative and thematic analysis the participants experience throughout their partners mental health issue is conceptualised in a Relationship Trajectory model. It illustrates the positive, early relational strength, superseded by relationship decline followed with relationship reinvention.

## Introduction

Military organisations are widely recognised as a deeply gendered institutions underpinned by structural and cultural norms, shaping expected behaviours and practices that influence how gender identities are performed within military communities [1,2]. Within this context, military spouses to serving/veteran military personnel inhabit a complex and often contradictory position. Despite being recognised as integral to the operational success and the retention of service personnel [3], the support provided by in military spouses is often marginalised and positioned at the fringes of military life [4–6].

### Military culture

Military enculturation presides over not just the serving person but also the family. The complexities imposed by military culture and the frustrations of military life felt by all involved are well documented [7–11]. Whilst military families face the same challenges that civilian families endure, they also must contend with a range of unique situations that other (civilian) families will never, or seldom face such as deployment, extended separations frequent relocations and inflexible work regimes [12]. Consequently, for military families, military life creates a culture that is unique to them.

Much of the extant research [2,13–15] have depicted military culture as hierarchical, disciplined, male-dominant and competitive, where dominance prevails and an expectation that ‘duty was meant to override love’ [16]. There is a growing consensus that suggests the military family (or family unit) is viewed as an integral part of the overall military community given that their private family life is synonymous with, and not separate to, military life. Values and beliefs underlain by stoicism, hierarchy, marriage, expectations, and mental health stigma of a military spouse have preceded to embody what military enculturation is today [1,17,18].

### Mental health within the military

Recent decades have seen an exponential growth of interest both politically and publicly regarding military conflicts, humanitarian operations and the resultant mental health of serving personnel [19–21]. For serving personnel and veterans alike, military studies recognise a range of mental health issues arising from combat and trauma exposure. For example, greater exposure to combat correlates with a deterioration of mental health and increased risk of suicide [22,23] and psychosocial stressors may also be associated with subsequent mental health issues. Previous research suggests that, irrespective of age, gender, rank, family/relationship troubles, and occupational problems, were the leading causes of depression in the armed forces [24]. Depression prevalence within military personnel is noticeably greater (23–26%) than that of the general population which is estimated at 13–15% [25,26]. Regardless, it is estimated that less than 40% of military personnel and veterans with a mental health issue will seek help [27] but may well require help from their family members and spouse.

### Mental health and military spouses

Limited evidence surrounds military spouses and their experiences of living alongside their serving partner with a mental health issue. There could be numerous reasons for this, including (but not limited to) global austerity measures, worldwide underfunding for the armed forces, and limited healthcare management models [26] have led to family members predominantly providing care to their military partner with a mental health condition [28]. Consequently, this may cause various and far-reaching psychological/adverse effects on those family members, especially the spouse [29] including divorce [30,31], care burden and impacts on health such as physical exhaustion, increased stress, anxiety and depression [32,33]. All of these are further compounded when considering the military context such as overseas deployments, working patterns, and military culture [34–36].

## Current study

The central aim of the study was to explore the lived experiences of military spouses of UK Armed Forces personnel who had experienced mental health issues, such as depression, anxiety, adjustment disorder, or PTSD. Existing literature had extensively explored the experiences of military families and deployment [6,37]; however, there was limited research specifically focusing on the mental health of serving personnel, the in-depth exploration of the lived experiences of their intimate partners and within the UK context. A systematic review was conducted to establish what was known about the lived experiences of military spouses whose partner has mental health issues. This yielded few studies, especially where the primary focus was on military spouses [38]. Thematic analysis of the 27 studies included in this review highlighted the importance of five core themes: Caregiver burden, Relationships, Psychological/psychosocial effects on the spouse, Mental health service provision and Spouse’s knowledge and management of PTSD symptoms [38]. The systematic review revealed notable scarcity of literature concerning spouses of serving military personnel and the unique cultural context in the UK. The review identified that although various methodologies were employed, no biographical approach was used and only a limited number of studies utilised interviews, which are essential for capturing the nuanced experiences of these spouses. Guided by the understanding that multiple realities exist [39], the study adopted an open and exploratory approach allowing for personal realities without the imposition of pre-existing labels or assumptions to be shared; thus, addressing the knowledge gap by illuminating the experiences of the UK military spouses.

To achieve the study, the objectives were refined to:

- investigating, through a biographical approach, the experiences of the military spouse whose UK serving military partner had sought support or treatment from mental health healthcare provision.
- explore the experiences of the military spouse during their UK military partners’ mental health issue and to better understand their role in this process and its effect on the relationship.
- explore their experiences to develop a deeper understanding of the challenges and enablers which help military spouse in their relationship with their UK serving military partner during a mental health issue.
- use the participant experience to develop a deeper understanding and make recommendations for future research and for practice, in supporting military spouse and the care of serving personnel with a mental health issue.

## Methodology

This study centred on the participant ‘their story, their voice, and their experience’ beginning from a starting point they selected themselves. Biographies whilst a novel approach in this research area and a first of its kind conducted within the UK context, afford the participants the freedom to share their story without restriction of time or place to uncover the idiosyncratic and collective aspects of living alongside a serving partner during their mental health issue. A biographical approach places an emphasis on the individual and the dynamic intricacies of their life; it enables them to share their own stories as perceived by them [40]. It allows space for those who are often marginalised or silenced a voice by positioning their individual experiences and their subjective explanation at the centre [41,42]. In most studies, military spouses are very often an adjunct within veteran or serving personnel studies [43–47]. Insight into their lived life story is gained by allowing the happenings and social circumstances that have shaped the various aspects of their lives to unfold [41,48]. A biographical approach acknowledges that lives are socially constructed and built up from numerous threads [49,50]. It moves beyond a specific snapshot in time, or a specific role allocated to the individual and illuminates an individual’s past and current experiences, their actions and their understanding, all of which have shaped their perceptions, their motivations and life course trajectories [40,51]. Storytelling affords the individual an opportunity to share and make clear to others, something about themselves and their untold story, permitting them to be seen outside of the roles undertaken (wife, mother, husband, father) [52]. Developing a sociology of stories from the sensitive perceptions of intimate personal experience from within the wider cultures and societies they inhabit humanises them from the darkness [41]. Including ordinary people’s stories of war, challenges dominant narratives and disrupt the assumptions made about women’s role in war and conflict and identifying military spouses at the heart of studies is a relatively new phenomenon [53–55].

### Feminist vision, values and spirit

Compelled by the sentiments of empowerment, equality and for the relationship to be one reciprocity, the collection of data in the present study aligned to feminist vision, values, and spirit [39,42]. The driving force was to understand the oppression of women, provide opportunities and voice to untold stories, to generate knowledge, to ‘make a difference’ and ‘create change’ [56; p.1]. In contrast to the notions of interview detachment, the interviewer-interviewee relationship is, first and foremost, the basis of the research with the presence of the researcher, as a person, acknowledged [42,51]. By doing so allows for more conversation (e.g., interactions and rapport building), which is fundamental when researching difficult and/or sensitive topics [57].

The preponderance of feminist research is that it is by women and for women [42]. In the present study, and understanding the military community, it was expected that female participants would predominate, however, it was accepted that all participants, regardless of gender, would share cultural commonality with one another [58].

### Researcher positionality

**Fig 1:**
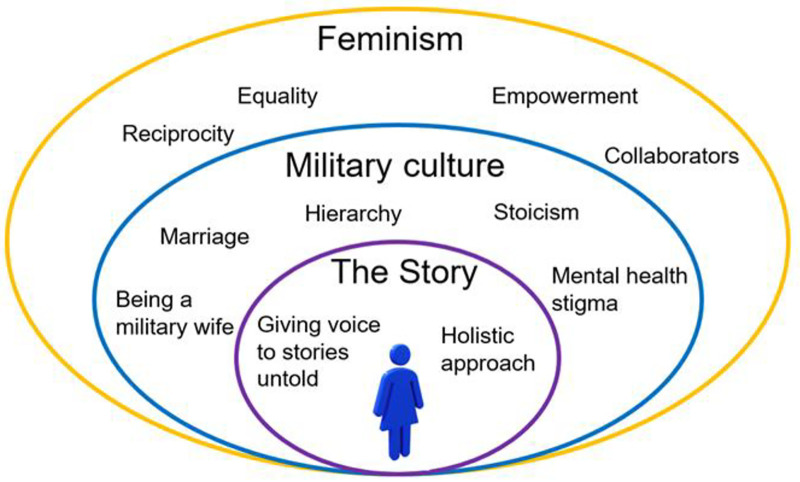
Conceptual thinking.

As a member of the military community (previous healthcare practitioner role with military and a military spouse) acknowledging the insider role was paramount [42,59], so that the conceptual thinking and the lenses in which we view the world highlight the standpoint of the researcher. The 1st author’s conceptual thinking identified three differing lenses as the foundation of making sense of the data: (i) the story; (ii) military culture, and (iii) feminism (Fig 1).

## Method

### Recruitment

A purposive strategy was adopted, and the recruitment strategy necessitated two approaches as it was recognised that there was a hard-to-reach element arising from ‘military culture, stoicism and stigma surrounding the sensitive topic. Initial recruitment employed a ‘non-invasive sampling strategy’ (n=4) using posters and postcards with study details and a contact email address. These were shared nationally with Community development workers who worked in collaboration with Military welfare teams. Service federations were contacted, and an article was published in the Army Families Federation magazine as well as a study promotion blog included on their social media feeds. Other military connected groups which included NSPCC mother and toddler groups, Military wives’ choirs, and coffee morning groups were approached to display posters and hold postcards for people to take away. This was followed by a purposive snowballing recruitment method (n = 6), common in hard-to-reach populations where participants referred new participants to the study [59]. The criteria for inclusion required participants who were a military spouse (inclusive of wife, girlfriend, husband, boyfriend, partner) of serving personnel of any rank across the tri-services, Army, Navy or RAF and that their serving partner had during their serving career experienced a mental health issue; namely, common mental health disorders (depression, anxiety), adjustment disorder, or post-traumatic stress disorder. Ten participants meeting the inclusion criteria came forward. Nine military spouses from across England and Northern Ireland participated in this study; one further participant referred through snowballing, on consideration declined to take part.

### The interview space

Data was collected between March 2018 and November 2019, over two separate in-person interviews at a venue and time of the participants choosing. The starting point of any biographical study is to engage participants either through sharing stories or joining alongside them as they live out their stories [60]. At the heart of the life story interview is the collaborative process of storytelling and listening facilitated by time spent together. Initiated from a broad, single narrative question ‘Tell me your story?’ the first interview provided a ‘biographical self-presentation’ [40]. With no starting point stipulated, for most participants, the stories began when they met their partners. Reiterating a sense of agency within the process, participants talked freely and without prompts or interruptions allowing them as much time as they wanted to talk about their experience [61].

To allow for follow-up and focused participant self-reflection, a second interview whilst more conversational in nature, was structured around four open-ended questions. This afforded greater focus specific to the study area namely: the participants own experiences thoughts and feelings. Opportunities for participants to revisit what they had said, occurred concurrently throughout both interviews by seeking clarification through pen portraits and summarising points made during the interviews [60,61]. To close the interviews, participants were invited to ask questions.

Participant information was issued ahead of the first interview, assurances of confidentiality and anonymity throughout the study were given and written consent was obtained from all participants prior to each audio-taped interview. Given the sensitive nature of topic, a debrief document and support document, identifying key agencies available for referral and support was given [42]. All the interviews were transcribed verbatim and stored in accordance with General Data Protection Regulations (2018).

### Ethical approval

Ethical approval was granted by the University Ethics Committee (Submission Ref: 2879). This study is reported following the Consolidated Criteria for Reporting Qualitative Research (COREQ) checklist (see supplementary information 1).

### Data analysis

The study adopted more than one analytical approach to allow different perspectives to arise from the data. Lieblich et al.’s (1998) framework was adopted for analytical pluralism whereby the completeness of the story and the generation of categories or themes are embodied. The framework offers two independent dimensions: ‘holistic versus categorical’ and ‘content versus form’ and assumes four potential avenues of data analysis however, it is noted that analysis need not simply fit into one [62; p12-13].

### Generating stories

Producing an authentic depiction of the participant and generate the final story a combination of ‘Holistic-Form’ and ‘Holistic-Content’ analytical approaches were executed. The transcripts were read and re-read prior to manually analysing the data for the stories. Whilst culturally and thematically different to this study, the three stages of the two-stage life story analytical approach was reflected within the participant stories and therefore adopted for this study. When asked to tell me their stories, a conjoined story about themselves, their serving partner and their relationship was shared, providing a context or rationale for their current situation. On analysis, the participants themselves were not always easy to locate in the story; the emphasis was very much focused on telling their serving partner’s story.

Generated initially from the stories was a collection of years and events which set the scene about their relationship. These events were collapsed into one and therefore were indicative of ‘*the beginning’* stage and named as such. The second part of the story telling was shorter yet more detailed specifically focused on ‘*changing times’* with the emergence and the duration of the mental health issue. ‘*This is me’* is the final stage of the story, illustrating the participants’ ‘*self*’ in this journey and current circumstances. The body of the story was then captured by applying a holistic-content analytical approach; a five-stage reading process allowing a temporally ordered description involving events, actions and interpersonal interactions within each stage. The final story was created by using raw data elements which were depicted in italics throughout the stories and narrative linkages drawn together from the data collected over two interviews. The couple’s story was captured from the participant’s viewpoint and as such, presents only a part of the overall picture. This was acknowledged through writing in the third person to differentiate from the stage ‘this is me’ where the data referred to the participant themselves. This was an important part of the analysis and is illustrated in the first person, ensuring the emphasis is on them and that they were not invisible within their partners’ stories.

### Generating categories

Generating the stories, meant that a deep familiarity with each of the transcripts. Unlike the manual strategy of analysis used for the stories, due to the amount of data collected NVIVO (version 12) was used to revisit the original transcripts. Within the framework, the ‘Categorical-Content’ elements are used to analyse and emphasize the interactions between insights arising from the individual stories and the themes emerging collectively.

**Fig 2:**
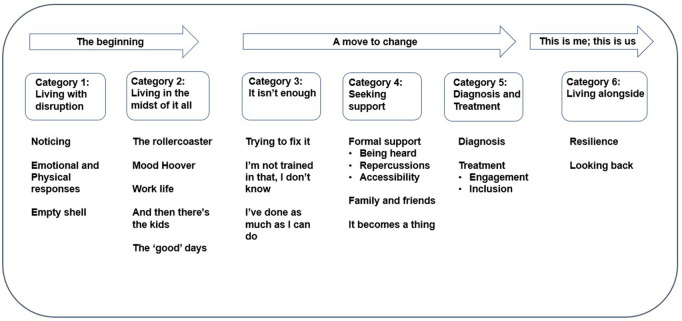
Category and sub-category illustration.

The approach exercised was subjective and involved the complex process of interpretation [62]. Unlike holistic individualistic analysis, categorical-content analysis collates and details the patterns from across the collective data set allowing for the comparison of structural elements along with identification of difference or similarities across the data [63]. The analysis began without any preconceived thoughts of what or how the subcategories would look like. In this study, the identification of utterances led to the subcategories (21) and then broader categories (6) (see Fig 2), which once identified by the first author, were critically discussed by the team.

Prior to any identification of words and texts, the aim of the study was revisited. The references made to any aspect of the experience of living alongside a mental health issue that captured something important such as a description of a behaviour, a feeling or response regardless of size or prevalence were highlighted. Such references materialised both spontaneously during the first interview and in response to the narrative generated questions used to deduce the participants’ experiences during interview two: ‘*On a good day, what is it like for you*?’

## Findings

During the analysis, a collection of demographic data was collated from each individual interview transcript and detailed in ref 1. The information drawn from the data, provides crucial context and understanding of participants individual characteristics as well as collectively. All data shared is in accordance with the participant’s consent, all participants and their family members have been anonymised using pseudonyms. Demographic data about each participant is detailed in Table 1.

**Table 1:** Participant demographic data.

Name	Recruitment method	Age bracket 18-25 26-35 36-45 46-55	Marital Status & relationship type	Relationship length: 0-5 years 6-10 years 11-15 years 16-20 years 21 + years	Accommodation type: Own home Private rented Military housing	Serving partner: Army Navy RAF	Serving Partner: Commissioned Or Other ranks
Christina	Non-invasive	18-25	Married Opposite -sex partner	6-10	Military housing	Army	Other ranks
Lindy	Non-invasive	46-55	Married Opposite -sex partner	21+	Own home & military housing	Army	Commissioned
Simone	Non-invasive	26-35	Married Opposite -sex partner	6-10	Military housing	Army	Other ranks
Lauren	Snowballing	26-35	Married Opposite -sex partner	6-10	Military housing	Army	Other ranks
Harry	Non-invasive	26-35	Married Opposite -sex partner	6-10	Military housing	Army	Commissioned
Cathy	Snowballing	36-45	Married Opposite -sex partner	11-15	Own home & military housing	Army	Other ranks
Sara	Snowballing	26-35	Married Opposite -sex partner	11-15	Own home	Army	Other ranks
Vikki	Snowballing	36-45	Married Opposite -sex partner	16-20	Own home	Army	Other ranks
Sophie	Snowballing	36-45	Married Opposite -sex partner	21+	Own home	Army	Other ranks

### The stories

The final stories originated from the data collected in both interviews; they give a nuanced in-depth insight into the experiences and are too vast for inclusion in this paper. Table 2 provides a vignette of each participant’s story. Each story shares an initial joint story where predominance was given to their serving partner and/or to provide context. These are represented in two stages: ‘*in the beginning’* and ‘*changing times’*. The final stage of the story illustrated by a vignette and representational quote in Table 2 focused on the participant themselves and was titled ‘this is me’.

**Table 2:** Participants vignettes.

**Christina**	*‘In a nutshell it’s hard, but you love them’*
Christina shared the challenges faced as a newly married couple, particularly the strain on their relationship due to her partner, Daniel, being in the military and dealing with his emotional instability. Christina was working in a care home and struggled with feelings of helplessness and frustration as she tried to support Daniel, especially during his deployments. Their marriage experiences significant ups and downs, with periods of happiness overshadowed by Daniel’s mood swings and mental health issues, including anxiety and depression. Christina felt torn between her commitment to Daniel and her own needs, especially when starting a nursing degree that limited her ability to support him during his struggles. Over time, she learned to balance her own aspirations with her role as a supportive partner. Despite the ongoing challenges, including Daniel’s counselling sessions and his fluctuating mental health state, Christina found strength in their relationship and began to assert her own needs. She acknowledged that while Daniel may not fully overcome his issues, their relationship has grown stronger as they navigate these difficulties together. Ultimately, Christina embraced her own identity and independence while remaining committed to supporting Daniel.	
**Lindy**	*‘I thought I bloody need a medal; there’s absolutely no recognition at all’*
Lindy reflected on her experience of marriage, feeling isolated and unsupported after moving away from home. She described a sense of loneliness and fear, exacerbated by a lack of connection with the local community and family. Despite living in the area since 2002, shame and gossip have prevented her from engaging socially, leading to resentment towards Craig, who prioritises military obligations over personal matters. Lindy expressed anger as she felt like Craig is an “empty shell”, she struggled with mixed emotions as they approached their 30th wedding anniversary, questioning the nature of their relationship. Efforts to communicate with Craig often felt futile, highlighting a deep sense of frustration and isolation. Lindy contemplated the impact of military life on their relationship and her own coping mechanisms, feeling that she had to adjust continuously without adequate support. Ultimately, Lindy expressed uncertainty about their future together and the possibility of change.	
**Simone**	*‘It is hard work, you can only do so much for them, it’s not easy at all’*
Simone expressed strong discontent with army life, feeling it negatively affected her partner, James. She disliked living in army quarters, finding the environment to be unsupportive of families. Initially, she thought James was fine, but after his return from Sierra Leone, she noticed changes in his behaviour, including increased drinking and anger, leading to frequent arguments. Despite her positivity and attempts to encourage him, their relationship became strained, and she began to feel depressed, seeking support from her parents. James was medically discharged and over time, she became more accepting of his bad days, trying to motivate him while also managing her own feelings of frustration. After James’s suicide attempt at Christmas, Simone felt forced to make decisions regarding his care. While James has started to open up, the couple still faced challenges with his appointments and their daily life. Recently, they have experienced more good days, but the situation remains difficult.	
**Lauren**	*‘I say this from my heart, I don’t think he would do it (take his own life), but I would hate it, the fact of I missed a sign because I would never forgive myself’*
Lauren reflected on her initial optimism about military life, which had now been challenged by the stress of her husband’s mental health struggles. Despite her love and desire to support him, she feels overwhelmed by the situation, especially as a mother of two small children. She worried about their safety and the impact of Damien’s mood swings and withdrawal on the family, which led to increased stress and a sense of isolation. Lauren expressed frustration over the lack of support from army welfare services, feeling their focus had been on the soldiers rather than families. She struggled to communicate her feelings to friends and family, fearing judgement and misunderstanding. The situation affected her patience and mental well-being, causing her to snap at her husband and contemplate leaving until he gets help. She felt helpless in understanding his mental health issues and was concerned about missing signs of deeper problems. The cycle of anxiety and medical emergencies further complicated their lives, leaving her frustrated and drained.	
**Harry**	*‘This is what you are like.....I’d tell Nicole every time when she was laughing, or she found something funny or was spontaneous or do something silly. I would always say that is, was nice to see her like this and just remind Nicole or just say well actually ‘this is what you are like!’*
Nicole’s army career had allowed her family to experience relatively little time apart, with only 13 months away over nine years. However, Nicole struggled with post-natal depression which created challenges that were hard for both her and Harry to remember clearly. Harry is a science teacher, he approached the situation with a practical mindset, focusing on ensuring the family is cared for while encouraging Nicole to seek help. Despite the difficulties, he maintained an optimistic outlook, highlighting moments of joy and spontaneity in Nicole to remind her of her true self. After the summer break, he returned to work, but the uncertainty of what awaited him at home weighed heavily on his mind. He often found himself concerned about Nicole’s well-being and the dynamics of their home life but would have never shared this with either friends or family. Although Nicole managed to perform daily tasks, her emotional struggles were evident, particularly during Sophie’s naptime. Harry expressed frustration over not understanding what triggered Nicole’s emotional responses, which made it difficult for him to support her effectively. Once Nicole began counselling, communication improved between them. She started sharing insights from her sessions, allowing for more open discussions about her feelings and experiences. This progress helped them both to engage more meaningfully in each other’s lives, enhancing their relationship amidst the challenges of mental health.	
**Cathy**	*‘This is so sad that for all these years our marriage has been tainted with this issue and actually it’s only now that we are the happiest, we have ever been’*
Cathy reflected on her struggles with post-natal depression and the strain in their marriage to Adrian, particularly during his deployment to Afghanistan. Initially, she accepted the challenges of his absence, but their unhappiness grew over time, exacerbated by Adrian’s reluctance to seek help for issues around sexual dysfunction. Despite feeling isolated and frustrated, Cathy remained in the marriage for the sake of their children and tried to create a fulfilling life independently. Cathy expressed disappointment with the army’s welfare system, feeling it was unapproachable for spouses. After reaching a breaking point, Cathy briefly separated from Adrian, leading to relationship counselling. Cathy believed that had Adrian been permitted by the army to seek help earlier, their marriage could have been better. Although their relationship is marred by these challenges, they now feel happier and are ready to engage more fully with family life, acknowledging the sacrifices made along the way.	
**Sara**	*‘It’s a real worry if something were to suddenly change’*
Sara reflected on her experiences with Tom, which began in childhood and continued into adulthood. When Tom returned home on leave from a tour of Afghanistan, he was intoxicated and angry about a family death that Sara had been asked not to disclose. After initially finding Tom’s behaviour amusing, Sara soon became concerned about his extreme mood swings, prompting them to seek counselling individually. Sara recognised that unresolved issues from her own childhood, particularly related to their father’s alcoholism and PTSD, had influenced her relationship with Tom. Sara described the anxiety of living with Tom’s unpredictable moods and the strategies she developed to manage conflicts. Sara noted that when Tom was in a good mood, life felt perfect and relaxed, but she remained apprehensive about potential changes. The couple decided against having more children to avoid further stress for Tom, opting instead for a dog. Sara acknowledged that she recognised Tom’s PTSD early on and chose to stay with him despite the challenges, often questioning her decision but felt a commitment to help him. Sara remains uncertain about the future, wondering if Tom’s issues might resurface.	
**Vikki**	*‘And I’m sat there thinking this might be funny to you, but this is my husband, and it’s our life. I’m the one getting the brunt of it’*
Vikki reflected on her experiences with their husband, Darren, after he returned from Iraq in 2003. Initially confused by his behaviour, she later recognised that he was struggling to adjust and remained on high alert. During a period when Darren sought medical help with the MOD, Vikki accompanied him to appointments, where she felt dismissed when a psychiatrist fell asleep whilst listening to Darren. Despite some happy moments, the situation worsened in 2016, leading to a tense atmosphere at home where even small interactions could trigger Darren’s anger. Vikki felt trapped and blamed for the issues, struggling to maintain their marriage while supporting Darren through his challenges. Vikki experienced anxiety over Darren’s mental state, particularly during distressing news reports, and she questioned her decision to marry him, acknowledging that they relied heavily on their supportive parents during this difficult time.	
**Sophie**	*‘But you know it’s better now, like it’s never going to be 100% ‘cos once it’s there, it never really goes away’*
Sophie reflected on the challenges of her relationship with Nathan, particularly after his return from Afghanistan in 2010. She took on significant responsibilities, leading to her independence, which Nathan perceived as him being unnecessary. As Nathan’s depression developed in 2012, due to being posted away married and unaccompanied, Sophie felt personally affected by his unhappiness and struggled with her own mental health. She often felt tense and frustrated, trying to understand his moods without him communicating his feelings. When Nathan received counselling after being signed off sick from his army role, Sophie felt excluded from the process, as she was not consulted about his treatment or outcomes. Although Nathan mentioned her depression, no one reached out to her to offer support, which she believed was inadequate. Sophie chose to avoid medication, opting for talking therapy instead, which helped her over time, especially after Nathan returned to work. Sophie acknowledged the ongoing challenges with her mental health but feels their relationship improved allowing for more relaxed and enjoyable days together, despite the continued stress and depression felt bu Nathan from his past military service.	

### The categories

Illustrated in Fig 2 are the final categories (6) and sub-categories (21) generated following the application of Lieblich et al.’s (1998) categorical- content analysis.

**Living with disruption** highlights the experiences of military spouses as they navigate the prolonged disruption in their relationships due to their partners’ mental health issues. Three subcategories emerge from this experience. Noticing illustrates the initial recognition of changes in behaviour. These changes varied from withdrawal and silence to anger and aggression. Some spouses observed these signs early on, while others, struggled to identify the changes due to their own emotional challenges. Responses to these changes ranged from confusion and paradoxical laughter to a gradual realization of the severity of the situation.

*…at first it was obviously quite funny, and I’d be laughing, but I would think to myself after, ‘ee god, what on earth is going on?” I didn’t really know at first. [Sara]*

The spouses described various emotional and physical manifestations of their partners’ mental health struggles, including night terrors, increased alcohol consumption, and hypervigilance. These behaviours often disrupted family life and created a tense atmosphere at home leaving participants feeling helplessness, lonely, and exhausted as they dealt with the unpredictability of their partners’ moods.

*He couldn’t function in a normal manner at all and then, it was more when he got back. He would wake me up in the middle of the night and his eyes would be open, but he would be asleep, and I remember him shouting at me; like ‘we need two helmets, we need them, we need them now’ and I’d be thinking ‘what the hell?’ [Sara]*

The emotional disconnect that arose from the partners’ mental health issues led some spouses to feel as though they were living with an “*empty shell*.” Communication difficulties and withdrawal from emotional engagement became pronounced, resulting in a sense of ambiguous loss for the spouses, who felt their partners were physically present but emotionally absent.

*“It was nothing obvious about Adrian, it was only that he never showed any form of any emotion from anything... I always knew that when he was with us that he would generally just sit and watch a lot, it was almost like, he needed to see everything, but for me I don’t know, it just felt a bit uncomfortable”. [Cathy]*

**Living in the midst of it all** captures the ongoing nature of these challenges, emphasizing the emotional rollercoaster experienced by spouses as they adapt to their partners’ fluctuating mental health.

*“I feel really gutted when I see that he’s in one of those, I don’t think it’s an episode, it’s a mood or whatever...gets that turmoil of emotion, that’s how I know when it’s coming and then I just sort of go in auto-drive…” [Christina]*

The themes of frustration, anger, and worry permeate their narratives, as they navigate the impact of their partners’ mental health on their own well-being and family dynamics.

*“It’s like when you are just at work and hear on the news about a train incident knowing what mood you have left them in and stuff like that, you don’t know if it is them or not. If they are that way and then they turn round and sort of say I would never be that stupid to do it, but you just don’t know do you?” [Vikki]*

**It isn’t enough** illustrates the point at which the participants realised their limits as well as their efforts to seek and receive help. The findings are categorized into three main themes. Participants often felt an inherent need to ‘fix’ their partners’ mental health issues, which stemmed from a blend of emotional commitment and a lack of knowledge. They expressed feelings of inadequacy regarding their ability to help, leading to a sense of frustration.

*[I said] ‘Well, just tell me what’s wrong?’ ‘I can’t fix it if you can’t tell me’. [Sophie]*

Many participants acknowledged their lack of understanding of mental health issues, which contributed to feelings of helplessness.

*“I thought, ‘I’m not trained in that, I don’t know’ and yet I knew there was something not right with him… This is the thing; I don’t understand it enough to be like ‘oh I understand why you are in a mood” [Lauren]*

This was sometimes overcome by the serving partner trying to reach out,

*“James showed me this video and he was ‘just like have a look at this’… he said, ‘just have a look at this video of this guy’. Eventually, this guy did kill himself ‘cos he just couldn’t cope anymore and he [James] said, ‘that’s how I feel, but that’s like how I feel’ and he had to do it through a video to show me” [Simone]*

However, there was also feelings of inadequacy after a prolonged period of trying to help. Some participants felt they had exhausted their resources, leading to tensions in their marriages. This prompted some to consider leaving their partners if they did not seek help, which in turn was often the motivation that led to the serving partners to seek external help.

*“I thought we were over… at the point now where I feel like I’ve done as much as I can do in the whole relationship, I wanted him to go to welfare, say that he is struggling mentally, I wanted him… and obviously eventually, he went to the doctor and sorted all that out” [Christina]*

**Seeking support** exemplifies the expressed frustration over the limited support available and reflected on their experiences with both informal and formal avenues of assistance, including consultations and treatments, and the varying degrees of involvement of military spouses in these processes.

Formal Support included medical and welfare services provided by the military. While some participants reported positive experiences,

*“The army have been good, they have supported him... he saw somebody and he had to go through like a decompression stage and then they do so many meetings after and I think they picked up something wasn’t quite right ‘erm and it was when I disclosed and said ‘look like he is having the bad dreams, like things aren’t quite right, like we have gone to the cinema and he’s cowering in the aisles’, ‘you gonna have to do something’, I said, ‘like this isn’t normal?” [Sara]*

Many felt that the military services focused solely on the serving personnel, often neglecting the needs of military spouses.

*“They all say like they are family orientated but they are not, they don’t think anything about families I don’t think, it’s all about them [serving personnel]” [Sophie]*

Once the serving partner had sought help, the participants faced challenges in being heard by the formal support systems. While some had positive interactions, many felt their concerns were overlooked or dismissed.

*“From a wife’s perspective, I don’t find them approachable, the whole system is not approachable ‘cos you have to, I can’t just rock up into camp, knock on welfare and say, ‘there’s an issue’, you know I can’t do that, it’s usually the serving member that has to go in and say, ‘there’s an issue”. [Cathy]*

Informal support systems were also evident; while some participants highlighted the strength they found from friends and family,

*“I don’t think I would have got through half of it, if it hadn’t have been for my mum and dad ‘cos I would just tip up at the door or ring them to say, ‘I’m coming over with the kids’ and they were like ‘yep that’s fine, not a problem’, you know” [Vikki]*

Whereas others were reluctant to share for fear of being judged.

*“The only people that you have to vent to, is friends but you can’t vent the real extent to them ‘cos you don’t want them to think that everything is rocky at home, ‘cos you don’t want them to think that, you want everything to seem ok, ‘yeah it’s not as good as what it was’ you can say but you don’t want to be ‘oh my god, they’re on the verge of divorce’ like you know, that’s a lot of it. It’s the same with family, you don’t want to tell them the true extent of it because otherwise, everyone’s very judgy, aren’t they?” [Lauren]*

**Diagnosis and treatment** of mental health issues often came later in the journey, after participants had already been living with the challenges for some time. Stigma both internalised and external along with the perceived hierarchy of mental health against physical health were all factors in this delay to seek help.

*“I’m talking relatively recently but we have recently experienced it [post-natal depression] but, if you go back, it wasn’t really talked about, really. I think we have friends that have got problems, that have proper injuries; I say proper, I mean physical types of injuries and things and then had to deal with those... I’d say from any officer, a lot of times, they don’t want everybody knowing about it and, there’s it going to welfare, the more people that find out it becomes a thing”. [Harry]*

The treatment varied, with many partners receiving counselling and medication. Participants noted varying levels of engagement from their partners in treatment, with some seeking external help independently.

*Couples therapy... “he’s just lying again and if he did it to me, that’s the biggest sin but then if you did it in therapy then your kinda not using it really (pause) and he has started doing to the therapist what he has done to me really, cancelling sessions because he would have work and he would do it at the last minute. At first, she did accommodate him and kinda did what I did and made excuses for him and then after a while, she stopped doing that” [Lindy]*

There was a notable lack of inclusion of military spouses in their partners’ treatment processes, which many participants felt could have aided their understanding and ability to support their partners.

*“I was quite surprised there was no kind of, [not] even one chat…I was surprised, I was surprised. I think I said to her at one point, and I was very surprised that I’ve had no formal involvement at all, absolutely nothing…The connection to the military wise, there’s been nothing really…” [Harry]*

**Living alongside** symbolizes the fluctuating journey between periods of their partner’s mental wellness and relapse, recognizing that the mental health issue was a constant presence. Strategies for coping varied among participants, with some focusing on accommodating their partner’s behaviours while others adapted their own responses to manage the relationship. Key themes emerged in the participants’ experiences, categorized into resilience and reflection on their journey.

*“You have to completely change your relationship anyway and you just have to either adapt or walk away…. he is not the person I met; he’s a lot different to who I originally, see ‘cos when I first met him, he’d never done a tour, but I was never gonna walk away because of my kids” [Cathy]*

Many participants developed protective interpersonal strategies, such as avoidance and accommodation, to cope with their partner’s mood swings and behaviours.

*“I suppose in order to survive it; I have had to adjust quickly all of the time, and I have become quite good at that... You have to have certain mentality ‘cos I think most people would just go…… I have accommodated him, and I suppose I am quite good at that” [Lindy]*

Over time, some participants noted personal growth and resilience, with changes in their own behaviour leading to improved relationship dynamics. For example, some shifted from merely accommodating their partner’s outbursts to actively challenging them, illustrating their development.

*“I don’t think Daniel’s changed at all since his initial treatment, I think I’ve changed, I think I’ve changed how I interact with him” [Christina]*

Participants also reflected on their experiences, often expressing feelings of heartache, resentment, and invisibility within their relationships.

*“I lived my whole life miserable so really, it’s been seven years of all that heartache and pain wouldn’t have been there” [Cathy]*

They acknowledged the toll that their partner’s mental health issues took on their own identity and sense of self.

*“I would say I am with this empty shell, and I have become more of an empty shell because of it” [Lindy]*

Despite these challenges, some participants recognized the strength gained from their experiences and the importance of their support networks.

*“You sacrifice a lot but then you gain a lot, the people I’ve met the network of the people I’ve got across the country is unreal, I’m really, really like I would never change that” [Christina]*

Both the stories and the categories indicate that living with a serving partner who has mental health issues is a complex journey marked by both struggle and growth. While participants faced emotional detachment and feelings of invisibility, they also identified gains in resilience and strengthened relationships.

### Relationship trajectory model

**Fig 3:**
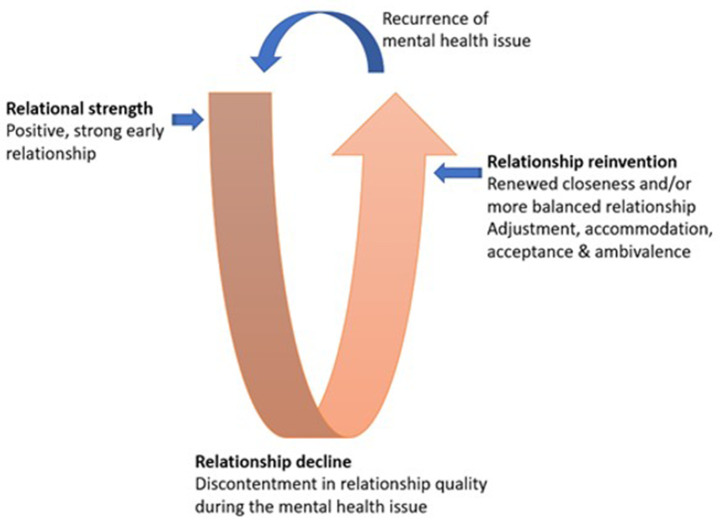
Relationship trajectory model.

Fig 3 illustrates the Relationship Trajectory model which conceptualises the coming together of the holistic and the categorical analysis. The data was not only interrogated separately but analysed together following the generation of stories and categories. While individual journeys varied, the majority followed the same trajectory, with military culture interwoven throughout. This led to the conceptualisation of the relationship trajectory model during one episode of a mental health issue. A predominant U-curve trajectory illustrates the participants’ experiences throughout the course of their partner’s mental health challenges; positive, early relational strength, followed by relationship decline and then a move to relationship reinvention during times of remission or effective treatment (see Fig 3). It was noted that this U-curve trajectory also applied during recurring episodes of mental health issues.

## Discussion

The central aim of the study was to explore the lived experiences of military spouses of UK Armed Forces personnel who had experienced mental health issues, such as depression, anxiety, adjustment disorder, or PTSD. A biographical approach facilitated the participants to share their lived experiences of living alongside a serving partner with mental health issues. Illustrated in the relationship trajectory model, the starting point of each story was meeting their serving partners, rather than from the point of their partner’s mental health diagnosis. **Relational strength** was emphasized through the sharing of relationship beginnings to provide context and highlight the bonds before facing mental health challenges. Whilst not a participation criterion, all participants were married aligning with findings [64,65] suggesting that military life encourages higher marriage rates to enable proximity to their partners. This study highlighted that the geographical and emotional distance from extended family, due to military postings, often led to stronger engagement and bonding between military couples, as illustrated in the stories of participants like Christina, Lindy, Lauren, and Cathy.

Irrespective of the factors preluding the mental health issue, participants noted a period termed ‘living with disruption’, characterised by prolonged behavioural changes in their partners that descended on a downward trajectory to **relationship decline** and subsequent discontent. After deployment, the reintegration process often mirrors a honeymoon phase but introduces unique emotional stresses [37,66]. Factors impacting relationships include feelings of abandonment during separations, resurfacing issues, and worries regarding the partner’s health, with many spouses concerned about latent mental health conditions manifesting upon their partner’s return [67].

Existing literature largely addresses relationships post-diagnosis of mental health conditions such as PTSD [68–71], neglecting the critical pre-diagnosis experiences of military spouses which explicitly highlighted the different ways in which their serving partners’ behaviours illustrated their emotional suffering well before any help seeking was initiated. This study found that spouses often interpret changes in behaviour as signs of emotional distress, and physical symptoms of distress emerge as triggers for concern [69–71]. Substance misuse, particularly alcohol, was observed among participants’ partners, reinforcing the connection between deployment experiences and relationship strain [72,73]. Communication within the relationship often falters, creating an emotional disconnect and difficulty in intimacy [47,69,74–77,78]. Complimenting Thandi et al.’s (2016) [77] findings, this study demonstrated the range of different physical and emotional intimacy experiences with no universal behaviour that is indicative of emotional disconnect between couples and a factor that contributed to the relationship decline.

A unique finding in this study, with no previous references made in military literature was the revisiting of images, media and war documentaries; a behaviour indicative of hyperarousal as suggested in studies outside of the military setting [79]. Possible factors for the lack of inclusion may be a less common behaviour or one that is overlooked and/or deemed acceptable because of the serving partner’s career choice.

Across the literature there is a predominate focus on negative connotations indicating that feelings of anger, anxiety, and frustration are common among spouses [74,76,80–83], however highlighted in this study was the extent and impact of how the serving partners mood both negatively and positively affected their emotional well-being and influenced their mood. Due to the unpredictability, the journey alongside their serving partner during their mental health issue was likened to ‘*being on a rollercoaster*’ or ‘*walking on eggshells’* the latter being the same idiom used in other studies [75,84].

Despite these challenges, many spouses demonstrated commitment to their partners, with a desire to ‘fix’ their issues. The motivation differs to previous studies which note dedication, loyalty and commitment to the relationship [47,69,74–76,80] which found the interventions made where driven by the lack of intimacy; a notable distinction from previous military spouse literature, which often emphasises caregiver roles and the burden associated with them. In the studies that prioritise relationship dynamics over caregiving roles, participants did not identify as caregivers, focusing instead on their marital relationships [80,84]. Notably, whilst the caregiving instinct was observed, none identified themselves explicitly as caregivers, reflecting a complex dynamic in their relationships and possibly the transitory nature of mental health issues where long periods of remission is noted. The findings are more akin to the maternalistic instinct demonstrated whereby there was an inherent, holistic need of trying to fix them [77,81]. Realisation of the inability to fix it, was then dominated with the sense of helplessness and uncertainty which is posited in care burden literature [33,85,86]. The biographical approach employed specifically illuminated these aspects among UK serving personnel spouses, revealing a gap in earlier research that presupposed caregiver roles through study aims and questioning.

Like other studies [6,37,66] acknowledging the impact of deployment through the application of the emotional cycle of deployment as a way of understanding the family unit during the post deployment period was evident however, this study also uncovers other stressors in military life, including operational demands and external factors like illness, which significantly disrupted family dynamics. Indicative of ‘non-deployment stress’ [87], this study emphasises that workplace pressures and demands resulting from a 24/7 availability requirement contribute to family stress, leaving military spouses/families feeling second to their serving partner’s career [16]. While findings resonate with existing literature [74,76–78,80,83,88] regarding mental health issues and relationship strains this study provides personalised insights into the experiences of participants, supporting previous studies’ conclusions.

Despite the raft of emotions and relationship discontent experienced, this study confirms a delay in help seeking, with military culture and both internalised and external stigma surrounding mental health acting as barriers. Partly driven by these cultural norms, in a move to protect the relationship, when the strain becomes too much, the findings suggest that the spouse are key enablers to serving personnel help seeking. These actions acknowledge and ultimately lead to addressing the discontent through help seeking across both formal and informal avenues of support, moving them forward onto a path of **relationship reinvention**. Consistent with current literature, when support was accessed, specifically the formal welfare/treatment provision, experiences were mixed, with some spouses feeling excluded from the treatment process for their partners [89,90]. Similarly to other literature [75,78,80], the findings underscore the need for greater inclusion of military spouses in therapeutic interventions and a re-evaluation of available support services, emphasizing the importance of addressing the unique experiences and needs of military families navigating these challenges.

This study highlights the complexities faced by military spouses living with a serving partner who have mental health issues, revealing both challenges and enablers that contribute to resilience within the marital relationship. Disruptions can threaten the relationship [33], so to aid the relationship reinvention, participants often reflected on positive aspects from the early days, which helped sustain their bond during difficult times.

This aligns with existing literature that notes military spouses cherish past positive experiences, particularly when their partner was mentally well [33,80]. Early positive couple functioning and the ability to recall and retain positive feelings from the early relationship was predominant in the findings and played a crucial role in coping with mental health challenges. Indicative of one of the levels within Couple Adaptation to Traumatic Stress (CATS) model is the ability to retain these positive feelings of couple functioning may go some way to explain how they overcame the bad days [91]. However, seminal literature surrounding non-military spouses’ highlights marital dissatisfaction and disruption persists even during times of remission [92,93] suggesting that military culture may also have an influence.

Consistent with other literature [30,47,69], participants reported using various protective strategies, such as avoidance and accommodation, to cope and ‘*survive*’ the day-to-day life with their partner’s disruptive behaviours. These strategies reflect the adaptability required in military life [6], although they can lead to long-term distress if overused [47]. This study underscores the ongoing emotional struggle faced by spouses but also highlights moments of acceptance and the potential for positive relationship growth despite the challenges posed by mental health issues. Ultimately, this study contributes to a deeper understanding of how military spouses navigate their unique circumstances, suggesting that a balance of meaning and satisfaction can be achieved even in the face of adversity.

### Strengths and limitations

A key strength is the diversity in ranks of the serving partners and their geographical spread, although the study is limited to only army spouses, therefore excluding the experiences of those from the Navy or Royal Air Force. Adopting a biographical approach and conducting in-depth life story interviews, is an original and novel approach which has facilitated the often marginalised, unheard voices of military spouses to be heard.

It is worth noting that the study’s small sample size and lack of diversity among participants—primarily female (n = 8) and all participants were married to opposite-sex partners—limit the scope of the findings. A broader and more heterogeneous sample, including more male and some same-sex spouses, could have provided additional insights into the experiences of all significant others of serving military personnel.

### Recommendations

The findings suggest a need for further qualitative research to explore common experiences among military spouses, emphasising their crucial role as informal support for serving personnel. They also emphasise the necessity for investigating support avenues for military spouses, such as peer-led education and information sharing, which are often sought informally rather than through structured support groups.

## Conclusion

This study represents the first in-depth exploration of the experiences of military spouses living alongside a serving partner with a mental health issue in the UK. Several contributions hold relevance in advancing both national and global understanding of military spouse experiences in the context of mental health. By adopting a biographical approach, the study amplifies the voices of military spouses and illustrates the complexity of their lived experiences, allowing for a nuanced understanding of their roles, challenges, and emotional landscapes, which had previously been underrepresented. Collectively the findings indicate that living with serving partners’ who have mental health issues is a complex journey marked by both struggle and growth. The uniqueness of this study lies in its focus on the period leading up to a mental health diagnosis, identifying symptoms and behaviours that, while similar to those found in post-diagnosis research, are present and concerning much earlier. This challenges existing literature by highlighting the prolonged emotional and psychological strain experienced by military spouses before any formal recognition of mental illness in their serving partner.

Aligned to existing literature in recognising both negative and positive experiences among military spouses, the study presents an original contribution by identifying how the serving partner’s mental health fluctuations ‘between good and bad days’ has a direct influence on the mood and emotional wellbeing of the spouse. This dynamic impact, observed before a formal diagnosis is made, adds a new dimension to understanding the emotional toll on military spouses and underscores the importance of early recognition and support.

In addition to what is known about the impacts on work life, this study offers alternative insight by describing the emotional pressure felt by the military spouse, caused by the worry and concern for their serving partner whilst at work.

During the treatment episodes, a contrasting finding to the existing knowledge was evident. Unlike the US studies, the study findings suggest military spouse inclusion and involvement in the management and treatment of the mental health issue was scarce. This invisibility was felt from both healthcare and welfare services. While participants faced emotional detachment and feelings of invisibility, they also identified gains in resilience and strengthened relationships. A further contribution to knowledge is the recognition of the whole journey trajectory conceptualised in the Relationship Trajectory Model.

## Supporting information

S1 File Supplementary information 1. This is supporting info (2).
(DOC)
